# Prevalence and Patterns of Prenatal Alcohol Exposure in Australian Cohort and Cross-Sectional Studies: A Systematic Review of Data Collection Approaches

**DOI:** 10.3390/ijerph192013144

**Published:** 2022-10-12

**Authors:** Sophia L. Young, Sarah E. Steane, Nykola L. Kent, Natasha Reid, Linda A. Gallo, Karen M. Moritz

**Affiliations:** 1 School of Biomedical Sciences, Faculty of Medicine, The University of Queensland, St. Lucia, QLD 4072, Australia; 2 Child Health Research Centre, The University of Queensland, South Brisbane, QLD 4101, Australia; 3 School of Health and Behavioural Sciences, University of the Sunshine Coast, Petrie, QLD 4502, Australia

**Keywords:** pregnancy, prenatal, alcohol, systematic review, surveys, questionnaires, interview, data collection

## Abstract

This study sought to determine data collection approaches in Australian cohort studies and explore the potential impact on reported prenatal alcohol exposure (PAE) prevalence and patterns. Inclusion criteria were that studies related to a general Australian antenatal population where PAE was assessed and reported. Studies were excluded if they were not peer reviewed, examined the prevalence of PAE in pregnancies complicated by alcohol-use disorders, or were published in a language other than English. A systematic search of five electronic databases (PubMed, Embase, CINAHL, Web of Science, and Scopus) was conducted. Risk of bias was assessed using the Effective Public Health Practice Project quality assessment tool. Results were synthesised using MetaXL. Data from 16 separate birth cohorts (*n* = 78 articles) were included. Included cohorts were either general cohorts that included alcohol as a variable or alcohol-focused cohorts that were designed with a primary focus on PAE. PAE prevalence was estimated as 48% (95% CI: 38 to 57%). When subgroup analysis was performed, estimates of PAE prevalence when self-administered surveys and interviews were used for data collection were 53% (95% CI: 41% to 64%) and 43% (95% CI: 28% to 59%), respectively. Use of trained assessors was an influencing factor of the prevalence estimates when data were collected via interview. Alcohol-focused studies reported higher prevalence of PAE, regardless of method of survey administration. Where interviewer training is not possible, self-administered questionnaires will likely provide the most reliable PAE estimates. No funding sources are relevant to mention. Review was registered with PROSPERO (CRD42020204853).

## 1. Introduction

Consumption of alcohol is culturally normative in Australia and, in 2019, almost one in three women reported alcohol use at some time during pregnancy [1]. Current Australian National Health and Medical Research Council (NHMRC) guidelines recommend abstinence when pregnant or planning a pregnancy [2]. Prenatal alcohol exposure (PAE) is a known contributor to intrauterine growth restriction, adverse organ development, and neurodevelopmental delay [3]. Preclinical studies have shown that even low levels of PAE are associated with the development of chronic disease, thought to be mediated via epigenetic changes [4,5,6]. Accurate identification of alcohol-exposed pregnancies is important to enable immediate antenatal and/or later family supports to improve child health outcomes, as well as facilitate the accuracy of clinical research findings.

Despite the known effects of PAE on birth outcomes and later life health [3,7,8], there is a lack of research exploring the prevalence of PAE and potential influencing factors in Australia. There have been national surveys and numerous pregnancy and infant cohort studies, including some focused on PAE as a primary outcome [9,10]. However, different data collection approaches have been used and this may have implications for accurate identification of alcohol-exposed pregnancies. It would be of immense value to compare study designs and data collection approaches and determine how these may impact prevalence rates and patterns reported.

The impact of data collection approaches on the reported prevalence and patterns of PAE has been examined in one Canadian study, whereby inconsistencies in the gathering of alcohol-use information across pregnancy cohorts was found [11]. There was disparity in the level of detail captured and the period of pregnancy examined. These findings resulted in a number of recommendations to promote a harmonised data collection method, to enable the future pooling of cohort data. A similar evaluation of PAE data collection methods in Australia may lead to context specific recommendations for a reliable and consistent alcohol assessment process, which could be used in future cohort studies and further clinical practice. The current review sought to investigate the influence of different data collection methods on reported PAE prevalence estimates in Australian pregnancy and infant cohort studies.

## 2. Materials and Methods

Reporting follows the Preferred Reporting Items for Systematic Reviews and Meta-Analyses (PRISMA) statement [12].

### 2.1. Eligibility Criteria

Studies were included if they met the following criteria: (1) cohort, cross-sectional, longitudinal, or case-control study; (2) majority Australian population (i.e., greater than 50%); (3) included an antenatal population; (4) prenatal alcohol exposure was assessed and prevalence was reported; and (5) the methods utilised for assessing alcohol consumption were provided. Government population-based surveys were included if results were reported in peer-reviewed publications. Studies were excluded if they: (1) conference abstracts, books, or PhD dissertations; (2) examined rates of maternal alcohol-use disorders (i.e., not assessing specific levels of PAE during pregnancy); and (3) were published in a language other than English. Studies were grouped by whether they were large (*n* > 1000) studies of a typical antenatal population, or smaller case and/or case-control studies (study characteristics and prevalence figures included in Supplementary Material Tables S8 and S9). Non-specific studies (those which did not specifically set out to examine prevalence of PAE) will herein be referred to as ‘general’ cohort studies, in contrast to ‘alcohol-focused’ studies.

### 2.2. Information Sources, Search Strategy, and Selection Process

Articles were identified from a systematic search of five relevant electronic databases (PubMed, Embase, CINAHL, Web of Science, and Scopus) from their inception until the 4 July 2022 for the keywords: “prenatal alcohol exposure”, “birth cohort”, and “Australia”. Supplementary Table S1 provides the full search strategy as applied to each database. The initial search and removal of duplicates was undertaken by author SY. Three independent reviewers screened the titles and abstracts (SY, SS, and NK) and full texts were reviewed by two authors (SY and SS).

### 2.3. Data Collection Process and Data Items

**Figure 1:**
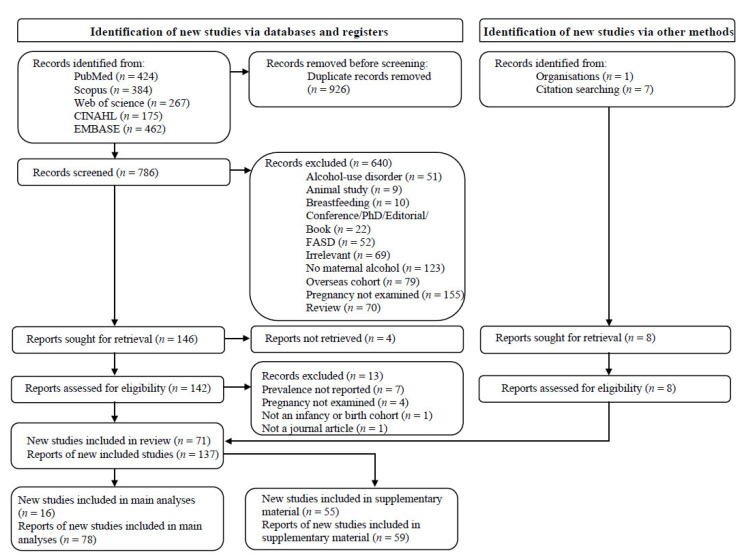
PRISMA flow diagram.

Data were collected from published reports by one reviewer (SY) and checked by a second where any findings were unclear (SS). As a requirement of study inclusion was that prevalence data were published, further prevalence data were not obtained or confirmed by study investigators. Information on questions used to capture alcohol consumption during pregnancy, as detailed for the Environments for Healthy Living (EFHL) study, Tasmanian Infant Health Survey (THIS), and the Barwon Infant Study (TBIS), were collected through contact with cohort study investigators. No automation tools were used in the data collection process. Relevant outcomes for which data were sought are listed in Table 1 and Table 2. Information regarding the number of women enrolled in the study, year of enrolment, year of pregnancy, availability of original questionnaire details, method of data collection (self-administered or interview), the period of PAE, details of alcohol consumed (occurrence/frequency/quantity), whether data were collected during pregnancy (prospective) or post-pregnancy (retrospective), the period of recall (i.e., entire pregnancy), and all available prevalence rates were extracted. Information from large cohort studies (>1000 participants) was tabulated, and those providing an overall prevalence rate of PAE were included in a meta-analysis. Small cohort studies (<1000 participants) and/or case-control studies underwent complete data extraction but were excluded from the main analyses (see Figure 1). Information was also sought on sociodemographic characteristics of included participants.

One paper from one additional cohort study was detected through personal communication (The Barwon Infant Study). Of studies included in the main analyses, data extraction was completed on 78 articles, 5 of which were identified through a reference list during full-text screening (Mater University Study of Pregnancy [13], Tasmanian Infant Health Survey [14], Tasmanian general cohort study [15,16] and the National Drug Strategy Household Survey [17]). Of studies included in the Supplementary Materials, data extraction was completed on 59 articles, 2 of which were identified through a reference list during full text screening (Lililwan [18], and everyday activities in pregnancy [19]).

**Table 1:** Cohort enrolment and method of data collection on prenatal alcohol exposure.

Cohort ^1^	Number Enrolled *	Year of Enrolment ^2^	Questionnaire Details Available	Method of Collection	Data Collection ^~^	Period Queried ^3^	Timing of Data Collection
PEMP [20]	7301	1975–1981	No	Interview	Prospective	Not specified	Not specified
MUSP [13,21,22,23,24,25,26,27,28,29,30,31,32,33]	8556	1981–1984	Yes	Interview	Prospective	14–26 weeks	19.8 ± 6.0 weeks gestation
						12 weeks	3–5 days post birth
Tasmanian cohort study [15,16,34]	56,037	1982–1989	Yes	Interview ^a^	Prospective	Not specified ^b^	Not specified
Victorian cohort study [35]	8884	1985	Yes	Interview ^c^	Retrospective	Entire pregnancy	At delivery
TIHS [14,36]	7945	1988–1995	Yes ^i^	Interview ^d^	Retrospective	Entire pregnancy	4 days post birth
RAINE [37,38,39,40,41,42,43,44,45,46,47]	2868	May 1989–Nov 1991	Yes ^e^	Self-administered survey	Prospective	12–20 weeks	16–20 weeks gestation (M: 18 weeks)
						Current use	34 weeks gestation
WAPIS (including RASCALS subset) [48,49,50,51,52,53,54]	4839	1995–1997	Yes	Self-administered survey ^f^	Retrospective	Entire pregnancy	12 weeks post birth
LSAC infant cohort [55,56,57,58,59,60,61,62]	5107	2004 (2003–2004)	Yes ^e^	Self-administered survey	Retrospective	Entire pregnancy	3–19 months post birth
ALSWH [63,64,65,66,67]	2132	1996 (2000–2009)	Yes	Self-administered survey ^f^	Prospective	Not specified	During pregnancy, not specified
NDSHS [17,68,69]	3281	2001–2010 (2000–2010)	Yes ^e^	Self-administered survey ^f^	Retrospective	Not specified	Within 12 months post birth
SCOPE [70,71,72,73]	1164	2004–2011	Yes ^g^	Interview ^c^	Prospective	Current use—15 weeks	15 ± 1 weeks gestation
						Current use—5 weeks	20 ± 1 weeks gestation
EFHL [74,75]	3351	2006–2011	Yes ^i^	Self-administered survey	Prospective	12–24 weeks	≥24 weeks gestation
Triple B [10,76,77,78,79,80,81]	1414	2008–2013	No ^g^	Interview ^h^	Prospective	0–12 weeks	0–13 weeks gestation
						0–12 weeks	14–25 weeks gestation
						0–12 weeks	26–39 weeks gestation
						8–20 weeks	8 weeks post birth
AQUA [9,82,83,84,85,86]	1570	2011–2012	No	Self-administered survey	Prospective	13–18 weeks	<18 weeks gestation
						7 weeks	25 weeks gestation
						10 weeks	35 weeks gestation
TBIS [87,88,89]	1074	2010–2013	Yes ^i^	Self-administered survey	Prospective	4–28 weeks	28 weeks
						Not specified	Post birth, not specified
Hunter New England Cohort [90]	1179	2017–2018	Yes ^j^	Interview ^k^	Prospective	12–37 weeks ^l^	12–38 weeks

* The number enrolled in the study may not be the number included in analysis. ^~^ Prospective indicates questions were asked during pregnancy, while retrospective indicates questions were asked post pregnancy. ^1^ References for all publications arising from each cohort indicated in brackets. ^2^ Year of pregnancy is indicated in brackets if different to year of enrolment. ^3^ This was approximated to time from appointment. PEMP = Pregnancy Environment Monitoring Program, MUSP = Mater University of Queensland Study of Pregnancy, TIHS = Tasmanian Infant Health Survey, WAPIS = Western Australia Pregnancy Infancy Survey, LSAC = Longitudinal Study of Australian Children, ALSWH = Australian Longitudinal Study of Women’s Health, NDSHS = National Drug Strategy Household Survey, SCOPE = Screening for Pregnancy Endpoints, EFHL = Environments for Healthy Living, Triple B: Bumps, Babies, and Beyond, AQUA = Asking Questions about Alcohol in Pregnancy, TBIS = The Barwon Infant Survey. ^a^ Interview administered by doctor, ^b^ Period not given, stated ‘early in antenatal period’. ^c^ Interview administered by midwife, ^d^ Interview administered by research assistant/research midwife at day 4 of life, ^e^ Interview details obtained via study website, not through research article. ^f^ Postal survey. ^g^ Partial detail provided for study questionnaire. ^h^ PAE interviews administered by trained assessor in person or over the phone, where preconception, T1 (pre and post awareness), T2, T3, and postpartum (8 week and 12 month) alcohol use were examined. Third trimester alcohol use was retrospectively assessed at the 8-week postnatal interview to capture exposure across the trimester. ^i^ Details were obtained through contact with authors. ^j^ The majority of questionnaire information was available, although some detail on wording was missing. ^k^ *n* = 1166 completed survey over the phone, *n* = 13 completed online. ^l^ Exact time period not given, although women recruited based on being between 12 to <38 weeks gestation, asked about alcohol use ‘since you found out you were pregnant’. The Hunter New England Cohort and NDSHS Cohorts from 2001–2007 were excluded from meta-analysis due to absence of overall PAE prevalence estimates. TBIS and NDSHS Cohort from 2010 were excluded from meta-analysis as while overall *n* > 1000, the overall prevalence of PAE was reported on for *n* < 1000 participants.

**Table 2:** Timing and Prevalence of Prenatal Alcohol Exposure.

Cohort	Prior to Conception					Prior to Pregnancy Recognition					During Pregnancy								
	Occurrence	Frequency/Quantity	Heavy	Binge	Prevalence	Occurrence	Frequency/Quantity	Heavy	Binge	Prevalence	Occurrence	Frequency/Quantity	Heavy	Binge	Prevalence Anytime during Pregnancy	Trimester	T1	T2	T3
PEMP	X	-	-	-	-	-	-	-	-	-	X	X	X ^a^	-	74.5%, 1.5% >30 g per day	-	-	-	-
MUSP	X	X	X	-	74.5%	-	-	-	-	-	X	X	X	X	49.6 %	X *	49.4%		35.3%
Tasmania (general)	-	-	-	-	-	-	-	-	-	-	X	X	X	X	40% (1982–1989), 55.9% (1981), 54.4% (1981–1982)	-	X	-	-
Victoria (general)	-	-	-	-	-	-	-	-	-	-	X	X	X	X	36.2%, 3.6% >50 g per occasion	-	-	-	-
TIHS	-	-	-	-	-	-	-	-	-	-	X	X	X	-	33.5%	X	28.8%	26.3%	25.9%
RAINE	X	X	X	-	-	-	-	-	-	-	X	X	X	-	47.7% ^b^	X *	45.4% ^c^		33.4% ^d^
WAPIS ^1^	X	X	X	X	79.8%	-	-	-	-	-	X	X	X	X	58.7% 28.2% throughout, 4.3% >50 g per occasion	X	42.1%	42.4%	45.6%
LSAC	-	-	-	-	-	-	-	-	-	-	X	X	X	-	37.6% infant cohort, 27.6% child cohort	X	25%	32.3%	33.9%
ALSWH	X	X	X	X	92.4%	-	-	-	-	-	X	X	X	X	75.7% ^e^	-	-	-	-
NDSHS (2001–2007) ^f^	-	-	-	-	-	-	-	-	-	-	X	-	-	-	34.9% (25–44%)	-	-	-	-
NDSHS (2010) ^g^	-	-	-	-	-	X	-	-	-	49%	X	-	-	-	20% after awareness	-	-	-	-
SCOPE	X	X	X	X	55.2%	-	-	-	-	-	X	X	X	X	38.3%, 10% >60 g per occasion	X *	38.3%	6.5%	-
EFHL	-	-	-	-	-	-	-	-	-	-	X	X	X	X	45.4% (34.8–53.2%) ^h^	X	- ^i^	33.7%	
Triple B	X	X	X	-	-	X	X	X	X	61.7%	X	X	X	X	62.2% ^j^, 36.94% after awareness, 15.5% binge prior to recognition	X	19.1% ^POST^	29.4%	29.6%
AQUA	X	X	X	X	79.1%	X	X	X	X	54.2%	X	X	X	X	58.7–61.4% 27% throughout, 18.5% >50 g on one occasion prior to recognition	X	30.9% ^POST^	31.9%	
TBIS	-	-	-	-	-	-	-	-	-	-	X	X	X	X	52.7%, 8% >50 g per occasion	X	35.9%	31.3%	-
Hunter New England Cohort	X	X	X	X	79.3%	-	-	-	-	-	X	X	X	X	14.2% post awareness	-	-	-	-

^1^ Including RASCALS subset. * Not all trimesters were examined. MUSP = Mater University of Queensland Study of Pregnancy, TIHS = Tasmanian Infant Health Survey, WAPIS = Western Australia Pregnancy Infancy Survey, LSAC = Longitudinal Study of Australian Children, ALSWH = Australian Longitudinal Study of Women’s Health, NDSHS = National Drug Strategy Household Survey, SCOPE = Screening for Pregnancy Endpoints, EFHL = Environments for Healthy Living, Triple B: Bumps, Babies, and Beyond, TBIS = The Barwon Infant Study, AQUA = Asking Questions about Alcohol in Pregnancy. ^a^ defined as >3 drinks per day. ^b^ Figure calculated using data in Table S1 of paper [37], *n* = 2804. ^c^ Women were asked at 18 weeks of gestation about alcohol consumption in the first three months of pregnancy. ^d^ Figures were from a subset of *n* = 2370, at 34 weeks. ^e^ Figures calculated to include lifetime abstainers (*n* = 163) that were excluded from overall study reporting. ^f^ *n* = 2462, questionnaire examined time period of ‘during pregnancy’. Figure based on average across 2001, 2004, and 2007 time periods. These figures are presumed to be after awareness [68]. ^g^ *n* = 819, questionnaire examined time period of before knowledge of pregnancy and after knowledge of pregnancy. ^h^ Figure calculated using data from both included papers, due to different years reported on. ^i^ While each trimester was examined, prevalence after the first trimester was provided. ^j^ Calculated from data in Table 1 [10]. ^POST^ Post-awareness of pregnancy figures are shown where applicable. Although findings from TBIS and NDSHS 2010 are detailed in the current table for data completeness, these cohorts reported *n* < 1000 and were excluded from meta-analysis.

### 2.4. Evaluation of Quality of Reporting, including Risk of Bias

The evaluation of quality of reporting was assessed independently by two authors (SS and SY), with discrepancies in scoring resolved through discussion and consensus involving a third author (NK). A modified version of the STROBE tool was used [91] (Supplementary Table S2). The modified tool incorporated questions from the Quality Assessment Tool for Quantitative Studies (EPHPP; Table S3) [92], due to the absence of a measure for risk of bias in STROBE. Additionally, a number of STROBE questions relating to ‘analysis’ were excluded (Supplementary Tables S2–S4). Supplementary Table S5 provides a summary of scores for each relevant paper. A maximum score of 47 across both measures could be achieved. Only publications included in the meta-analysis were evaluated. A quality score of 20 and below was considered low quality, 21–35 was considered medium quality, and 36–47 was considered high quality.

### 2.5. Effect Measures and Data Synthesis

Prevalence estimates were used in the synthesis and presentation of results. Studies were eligible for inclusion in the synthesis if overall PAE prevalence was reported. Inclusion also relied upon data being reported for *n* > 1000 participants. Where prevalence of PAE within a cohort was reported in more than one publication, the prevalence reported by the study that included the largest number of participants was used to generate the pooled estimate and is referred to as the primary paper. If two articles presented information from different time points for the same study, data were manually combined from the relevant publications to give an overall prevalence value. Meta-analysis was performed for synthesis of results. The excel software plug in MetaXL (Version 5.3, EpiGear International, Queensland, Australia) was employed to calculate a pooled prevalence estimate for PAE from the large cohort studies identified, and to produce a visual display. The quality effects model was used, where weight calculations were based on the quality score and sample size of the primary paper [93]. I^2^ values provided an indication of heterogeneity. Subgroup analysis of studies collecting data through interview versus self-administered survey was used to explore possible causes of heterogeneity. Sensitivity analysis was performed. The Luis-Furuya-Kanamori (LFK) index was used to quantify Doi plot asymmetry, a possible indicator of publication bias [94]. Confidence intervals are reported.

## 3. Results

### 3.1. Search Results

Searching of relevant bibliographic databases identified 1712 records (Figure 1). Following duplicate removal, title and abstract screening of 786 unique articles identified 146 eligible studies for full-text screening, of which 129 studies met the inclusion criteria. Thirteen studies were excluded at the full-text screening stage, primarily due to prevalence not being reported (Supplementary Table S6). Seven additional studies were identified through searching of reference lists of included studies, and one additional record [87] was identified through personal communication. Data extraction was completed for all 137 records. Findings from 16 different large cohorts (>1000 participants) were reported by 78 publications, from which all relevant details on study design and PAE were extracted to tables (Table 1 and Table 2, Table S7). Meta-analysis was completed on the majority of these studies. Absence of overall PAE prevalence estimates resulted in the exclusion of the Hunter New England Cohort and the NDSHS Cohort from 2001 to 2007 from meta-analysis. Where data for overall prevalence were not reported for *n* > 1000 participants (The Barwon Infant Study and NDSHS Cohort from 2010), these results were also excluded from the meta-analysis. Findings from 55 small cohort studies (<1000 participants) and/or case-control studies were reported by 59 publications, from which all relevant data were extracted and tabulated (Supplementary Tables S8 and S9) [18,19,95,96,97,98,99,100,101,102,103,104,105,106,107,108,109,110,111,112,113,114,115,116,117,118,119,120,121,122,123,124,125,126,127,128,129,130,131,132,133,134,135,136,137,138,139,140,141,142,143,144,145,146,147,148,149,150,151,152].

### 3.2. Risk of Bias and Quality of Included Studies

No studies were considered low quality. General cohort studies were considered medium (11 of 14) or high (3 of 14) quality (Table S5). The two alcohol-focused studies [9,10] were rated as high quality (Table S5).

### 3.3. Meta-Analyses

**Figure 2:**
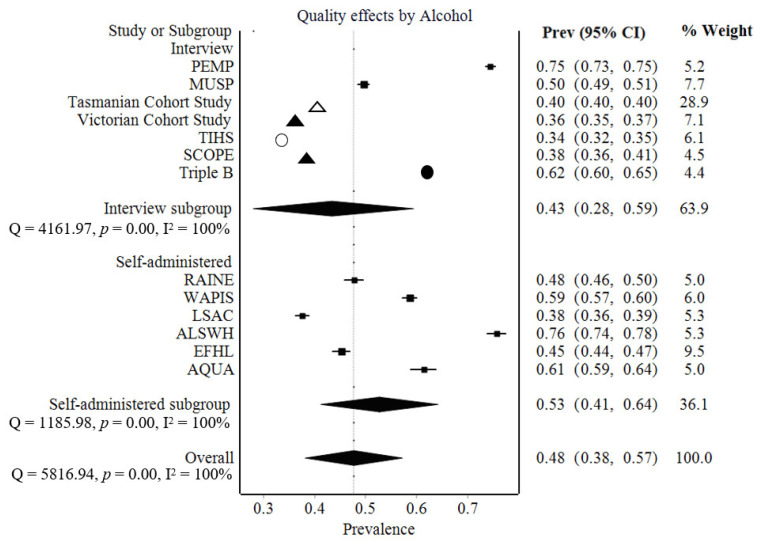
Forest plots displaying prevalence estimates with 95% confidence interval (CI) and subgroup analysis for alcohol exposure at any time during pregnancy for cohorts in which information on PAE was collected using self-administered survey-based methods and interview-based methods. I^2^, between study heterogeneity; Q, Cochran’s Q; *p,* statistical significance. For each cohort, the prevalence rate reported by the publication using the largest sample of that cohort is shown. An overall prevalence value was not given for the NDSHS 2010 cohort, where the value prior to pregnancy recognition was used. The Triple B study used trained assessors to collect interview information. The Hunter New England Cohort and the NDSHS Cohort from 2001 to 2007 were excluded due to only reporting prevalence after awareness. The NDSHS Cohort from 2010 and the Barwon Infant Study were excluded due to small sample size reporting overall prevalence (*n* < 1000, despite overall cohort size *n* > 1000). Triple B and AQUA cohorts differentiated between pre- and post-recognition of pregnancy. For the interview subgroup, open triangle represents doctor, closed triangle represents midwife, open circle represents research assistant/research midwife, and closed circle represents trained assessor. Boxes in the interview subgroup represent studies where the assessor was unclear. Results suggest that while overall self-administered surveys generated higher prevalence estimates than interview-based methods, and the use of trained assessors in interview settings resulted in high PAE estimates.

Considerable heterogeneity existed between studies (I^2^: 100%). Subgroup analysis was completed to investigate this, as shown in Figure 2. High heterogeneity remained, with both subgroups recording an I^2^ value of 100%. Analysis of pooled prevalence rates across all studies resulted in an LFK index of 3.56, representing major asymmetry in the Doi plot, which may indicate publication and related bias. Sensitivity analysis was completed, and results appeared robust, as shown in Supplementary Table S10.

### 3.4. Study Participants and Summary of Alcohol-Use Assessment Methods

Of the 16 large cohorts examined, 15 included between 1000 and 10,000 enrolments, with 1 [34] including 56,037 pregnant women (Table 1). Studies were typically between 1- and 3-year duration, and collectively spanned 1975 to 2018. Of the 16 cohorts, 2 had an alcohol focus (i.e., AQUA [9] and Triple B [10]). The majority (13/16) had details of the original questionnaire used to ascertain alcohol use available. Eight studies collected alcohol use data through interview conducted by: a doctor [34], midwife [35,72], research assistant/midwife [10,14], computer assisted [90], or the assessor was not reported [13,20]. One study used trained interview assessors [10]. Eight studies used a self-administered survey. Alcohol consumption prior to conception was collected by nine of the cohort studies (Table 2). Alcohol consumption prior to pregnancy recognition was examined in three cohorts, two of which were the alcohol-focused studies. While all cohorts collected information on the occurrence of alcohol consumption during pregnancy, and most on the frequency and quantity, the occurrence of binge drinking was less commonly captured (11 of 16). Ten cohorts included questions on trimester-specific drinking habits. In 11 studies, the women were interviewed or administered the survey prospectively during pregnancy, in comparison to 5 where PAE was retrospectively collected post-pregnancy. The majority of studies where PAE data were collected during pregnancy had a period of recall between 12 and 18 weeks, with four having a possible recall period of 20 weeks or greater [37,75,87,90].

### 3.5. Patterns of Prenatal Alcohol Exposure

#### 3.5.1. Timing and Prevalence of Alcohol Exposure

Prevalence estimates of alcohol consumption across pregnancy were reported by 15 large cohort studies with meta-analysis yielding a pooled prevalence estimate of 48% (95% CI: 38 to 57%; Figure 2). However, there was a wide range in prevalence estimates between studies as indicated by the I^2^ value, suggesting the presence of subgroups (Figure 2). Given that the overall prevalence of PAE varied based on whether the method of collection was self-administered survey (45–76%, with one cohort estimate 38%), or interview (34–63%, with one cohort estimate 75%), subgroup analyses were performed. Cohorts using self-administered surveys resulted in a higher pooled estimate of PAE prevalence (53%, 95% CI: 41 to 64%; Figure 2) compared with those using interview-based methods (43%, 95% CI: 28 to 59%; Figure 2); however, high variability between studies within subgroups remained as indicated by I^2^ values. For cohorts using interview-based methods, the forest plot suggested that one source of this heterogeneity may be the assessor conducting the interview; trained assessors were found to yield higher estimates, which more closely resembled those obtained from self-administered questionnaires. For both subgroups, other potential sources of variation in the estimates of PAE prevalence between cohorts were the prenatal period(s) assessed and the timing of assessment relative to exposure. Prevalence of alcohol consumption during pregnancy varied from 33.5% [14] to 75.7% [63] but was often reported between 40% and 60%. While most studies did not differentiate between prior to and post-recognition of pregnancy, three studies [9,10,68] consistently reported between 50% and 60% prevalence prior to pregnancy recognition. These same studies reported between 20% and 40% prevalence post-recognition. Prevalence also differed across trimesters, with lower ranges usually reported for the first and second trimesters. Although the frequency reported from both prospective and retrospective collection was variable, questioning prospectively during pregnancy was generally associated with a higher average percentage (38–76%, with 9/10 cohorts at or above 40%) than retrospective collection (34–59%, with 3/5 cohorts below 40%). Time period when the study was conducted did not appear to influence prevalence ranges, although two studies [68,75] noted a steady decline in self-reported PAE across subsequent recruitment years.

#### 3.5.2. Frequency and Quantity of Alcohol Exposure

Despite high prevalence, most studies reported a low level of PAE in pregnancy. Studies that did not distinguish between prior to and post pregnancy awareness commonly reported ranges of one to two drinks at no more than one to two days or sessions per week. In studies that documented binge consumption, prevalence was between 5% and 10%. In studies that examined alcohol use in the period prior to pregnancy recognition, almost 1/5 (16–19%) reported binge levels [9,10], although this did not persist post-pregnancy awareness. There did not appear to be a relationship between method of data collection (interview or self-administered survey) and the reported frequency and quantity of PAE, although this could not be compared quantitatively.

#### 3.5.3. Findings from Smaller and/or Case Control Studies

Of the smaller and/or case-control studies examined (Supplementary Tables S8 and S9), prevalence ranges were more variable (0–77%) and tended to be lower, particularly when data collection was through routine clinical appointments (1–23%). However, many of these studies examined specific populations that may be less likely to consume alcohol or report alcohol exposure (i.e., adolescent pregnancy, assisted reproduction, or where the relationship to other sensitive maternal or infant health conditions were being examined). When these smaller studies included a control population (*n* = 11), the ranges of PAE in the control group were sometimes similar to those of the larger cohort studies included in Table 1 (40–60%), although they were still variable. Very few smaller studies examined the period prior to pregnancy recognition, and so it could not be discerned whether these ranges were specific to pre- or post-recognition. Two of the more recent studies, however, did examine this time period. Prevalence of PAE pre-awareness was between 76% and 77%, and post-awareness (trimester 3) was between 12% and 24% [128,129].

## 4. Discussion

Profiling of PAE is critical to the design and implementation of effective antenatal and postnatal family supports for the prevention of alcohol-related harm. There have been two alcohol-focused cohort studies in Australia, and both reported a relatively high level of PAE (60–70%). In contrast, in the general, non-alcohol-focused cohort studies, PAE prevalence was more variable and often up to 30% lower, consistent with the most recent national survey data [1]. In the current review, we found that the use of self-administered questionnaires, rather than interview-based methods, to collect PAE data in general cohort studies tended to reveal higher prevalence estimates that more closely matched those reported by alcohol-focused studies. However, in one of the alcohol-focused studies where interviews were conducted by trained interviewers as opposed to healthcare professionals, PAE was higher and matched estimates from self-administered questionnaires. In addition, the timing of the questionnaires also influenced prevalence estimates, with prospective questioning eliciting a higher range of prevalence estimates compared with retrospective questioning. This highlights the importance of data collection approaches and provides impetus for the development of a consistent and reliable framework for the collection of PAE information across Australian cohort studies and, critically, in clinical practice.

Reduced prevalence ranges of alcohol consumption in interviews with untrained assessors compared to self-administered questionnaires could be due to perceived stigma, fear of judgement, and/or concerns regarding anonymity or confidentiality of information [153]. A drawback of large general cohort studies using interview-based collection methods may be poor availability for continuing staff (in longitudinal studies) or insufficient time to build an adequate connection with the participant to facilitate accurate reporting of information. In contrast, the increased prevalence estimates gathered in studies using trained assessors could reflect the quality of the information provided and the abilities of the assessor to ask the questions in a sensitive and supportive manner While limited research exists on healthcare workers’ perceptions of occasional alcohol use in pregnancy, some antenatal care providers of women with substance use issues have been shown to have negative or judgemental implicit attitudes towards their patients [154]. Negative and judgemental attitudes likely contribute to a fear of repercussions and may act as barriers to open discussion of alcohol use in pregnancy. Staff inconsistency and a poor level of support (low visit frequency, short appointment duration) may also be potential barriers [153]. Continuity of care has been shown to encourage trust and open communication between patients and their healthcare provider [155,156]. Although not specifically considered in the included studies, the quality of the relationship with the health professional may have negatively impacted reporting accuracy from interviews [157,158,159,160].

In the current systematic review, we reported high variability in estimates gathered through interview. This was largely due to relatively high prevalence estimates in two of the cohorts. While one of these was an older study and findings were likely reflective of that time period [20], the more recent study noted its use of trained assessors [10]. In the Triple B study, data were primarily collected through face-to-face interviews, and participants provided with images of standard drinks for varying types of alcohol. Interviewers were trained, experienced in assessing substance use in pregnant populations, and emphasized the importance of honesty with regard to the research questions. While it was not possible to test for alcohol in urine, biological testing for other substances in the Triple B cohort study showed accuracy of reporting at close to 100%. Consequently, in large general cohort studies examining antenatal populations, PAE prevalence is likely to be more accurately determined using self-administered surveys. However, where appropriately trained assessors are available, interview-based methods could be equally as effective.

There was large variability in the questions asked around alcohol consumption, particularly whether binge alcohol consumption was examined. Specifically, 11 out of 16 cohorts collected details regarding binge exposure. This is notable given that binge exposure has a greater risk of detrimental outcomes during pregnancy because it results in high blood alcohol concentrations, compared to a lower consistent pattern of drinking [161,162]. Additionally, while the large studies commonly examined frequency and quantity of alcohol exposure, this was not typical of the smaller studies, which often assessed alcohol as a binary outcome (i.e., yes/no). Another important consideration was the inclusion of questions that enable the comparison of data from pre-pregnancy recognition and post-pregnancy recognition. Overall, 1 out of the 14 general cohort studies (not including the 2 alcohol-focused studies) included information on alcohol exposure pre-pregnancy recognition; however, the level of alcohol consumed was not examined. Notably, two of the more recently conducted smaller cohort studies examined these periods, and generated estimates similar to the alcohol-focused studies [128,129]. Considering the potential impacts of PAE on birth outcomes, and the known influence of both timing and exposure threshold, the variability in data collection methods may limit the clinical relevance of findings [51,163]. While likely adequate to examine gross differences in birth outcomes due to PAE, general cohort studies may be unable to draw out the more nuanced potential impacts of PAE. This may also speak more broadly to the often conflicting findings regarding the potential positive effects of low levels of PAE [164], which have frequently been attributed to differences in sociodemographic characteristics, but may also be related to differences in pattern, dosage, or timing of PAE. Interestingly, the finding that the decade of recruitment did not impact prevalence range was despite a reduction in the overall consumption of alcohol in the community across the examined time period (1975–2018) [165].

Incongruity across data collection methods has been observed internationally. A previous review of Canadian pregnancy cohorts noted analogous challenges in the gathering of consistent alcohol-use information [11]. Although it would be ideal for pregnancy and infant cohorts to examine PAE at the level of detail seen in alcohol-focused studies, this would rarely be practical. Consequently, the importance of a ‘midpoint’ in data collection methods should be examined. As recommended by Poole et al., incorporating aspects of the AUDIT-C, such as frequency of use (never, monthly or less, 2–4 times a month, 2–3 times a week, or 4 or more times a week), quantity of alcohol consumption on a typical occasion (1–2, 3–4, 5–6, 7–9, or >10), and frequency of ‘at-risk’ binge drinking occasions (monthly, less than monthly, weekly, or daily or almost daily) would likely be the most appropriate approach [11]. The inclusions of a picture reference chart, questioning changes to alcohol consumption following recognition, and ascertaining a ‘quit-date’ would likewise increase data utility [11]. The use of a pictorial could also assist with calculation of a standard drink, which would be particularly important if an attempt were to be made to compare between international studies. Additionally, findings from the AQUA cohort suggest inclusion of a ‘special occasion’ drinking question to capture episodes of binge consumption that otherwise may not have been reported [9]. Including a minimum set of questions to capture the timing and level of alcohol exposure, as well as binge episodes, across the four periods of pregnancy (prior to recognition, trimesters 1–3) would likely elevate consistency and allow for aggregation of data across future cohort studies. In our review, we identified that the Western Australian Pregnancy and Infancy Study (WAPIS) cohort used retrospective, self-administered data collection methods and gathered detailed information including exposure prior to conception and timing of exposure during pregnancy. The WAPIS study reported PAE prevalence of up to 58.7–63.9% at any time and 37.2% throughout [48], which closely aligns with prevalence estimates from the alcohol-focused studies [9,10]. Thus, the findings of the WAPIS study highlight the utility of including detailed, self-administered questionnaires in general cohort studies where trained assessors or validated assessment tools may be unavailable.

### 4.1. Limitations and Future Directions

The identification of relevant studies was dependent on ‘alcohol’ being included in the title or abstract, and the exclusion of grey literature. This may have led to omission of several non-alcohol-focused, yet relevant, studies and limited our analyses to those with a higher quality of alcohol-related data. The inclusion criteria also resulted in the exclusion of more recent data produced by the Australian Institute of Health and Welfare as part of the National Drug Strategy Household Survey, as this data had not been published in a peer-reviewed paper. Results from the 2019 survey placed prevalence of PAE prior to recognition at 54.9%, and while pregnant as 29.7% [1]. Other limitations inherent to each included study should be noted, including homogeneity of study participants. Studies that detailed Indigenous population had rates comparable with the general Australian population, of which an estimated 3.3% identify as Indigenous [166]. As a whole, the included studies were generally not representative of private patients, those living in rural areas, or individuals not fluent in English. These populations were not specified in search terms, owing to the focus on ‘general’ cohort studies. Thus, PAE prevalence in Indigenous, rural, and culturally and linguistically diverse groups have not been sufficiently captured in the current review.

Studies with an alcohol focus were presumed to provide the most accurate estimate of PAE. However, this is an assumption based upon their typically higher reported prevalence ranges and extensive data collection methods. Validity could be determined through testing of alcohol exposure through biological samples, which was not a component of any of the included studies (alcohol-focused or general studies), although as noted earlier, the Triple B cohort reported high consistency of estimates for other substances. These alcohol-focused studies were conducted in defined, low-risk pregnancy populations (i.e., metropolitan public health service in Victoria, New South Wales, and Western Australia), and thus, while likely representative of the general urban population, may not be representative of women at higher risk of alcohol use during pregnancy.

Prevalence of prenatal alcohol exposure is acknowledged as being higher in advantaged populations. However, the quantity of alcohol exposure is typically significantly lower. The two alcohol-focused studies included in the current review reported on such populations, which may have resulted in artificial inflation of estimates. Additionally, the motivators for PAE in low and high sociodemographic groups may vary. Particularly in disadvantaged community groups, there is also likely to be the influence of poverty, past and ongoing stress, and trauma predisposing and perpetuating PAE. These drivers of PAE may determine which data collection tools are most appropriate. It would be beneficial for future studies to consider the role of SES and other contextual factors in influencing PAE, and to report specific the sociodemographic characteristics of included participants.

Finally, the usefulness of more modern methods to track PAE, such as smart devices, was not examined. The included studies that used self-administered surveys collected data through either paper-based or online methods. These burgeoning app-based techniques have the benefit of easy adaptability and can offer both a visually appealing and interactive interface. Accordingly, this option may combine the benefits of both survey and interview-based data collection approaches. A study of Aboriginal and Torres Strait Islander Australians has already validated one such alcohol recording tool, the Grog Survey App [167]. The applicability of this device to other cultural groups has not yet been established but may represent an area for further investigation.

In terms of review processes, analyses demonstrated significant heterogeneity and the level of publication bias remains unclear. The cause of heterogeneity, as evidenced by the high I^2^ value, may be due to differences in methods of data collection (including type of interviewer where interview-based methods were used), the period of pregnancy assessed, and the timing of assessment relative to exposure. However, validity of I^2^ for exploration of heterogeneity in systematic reviews of prevalence has been questioned, with a recent systematic review noting that upwards of 75% of systematic reviews of prevalence reported an I^2^ value of 90% or more [168]. I^2^ value may misrepresent heterogeneity in meta-analyses of large studies with precise confidence intervals, as relevant to prevalence reviews. Fortunately, high heterogeneity is unlikely to translate to imprecision in the prevalence estimate [169]. While a high LFK value was present for pooled studies, which may indicate publication bias, this is only an indicator and better reflects the asymmetry of included publications. Figure 2 demonstrates that studies with lower precision were typically placed to the right of the pooled estimate, indicating higher prevalence. Thus, findings of high heterogeneity may be more related to methods of data collection than publication bias. Asymmetry was reduced when subgroup analyses were performed, suggesting these findings may provide more appropriate insights into the variability of PAE estimates with data collection methods.

### 4.2. Implications for Future Research, Clinical Practice, and Policy

The current study highlights the importance of data collection methods in attaining accurate estimates of PAE, with implications for future cohort studies. For general cohort studies aiming to gather PAE information, the inclusion of self-administered questionnaire-based methods, which includes consideration of frequency, quantity, and binge type exposure and can differentiate pre-pregnancy, pre-recognition, and during pregnancy time points, could allow for more consistent prevalence estimations. However, where assessors trained in PAE data collection are available, interview-based methods could result in similar prevalence estimates. Ideally, results from the current study may inform the development of a universal PAE questionnaire, which could be utilised across Australian pregnancy and infant cohort studies. While one was proposed in 2010 based on in depth feedback from relevant focus groups [170], this draft questionnaire was relatively long and has not been widely adopted.

Estimates of PAE gathered from routine clinical practice appear similar to those collected from general cohort studies that also used interview methods. Indeed, a recent study noted that between 30% and 40% of pregnancies were prenatally exposed to alcohol across the 2013–2020 period [171]. Overall, however, these estimates are lower than those collected from research studies (~40–60%), and it was noted that data were missing for approximately 50% of the population each year [171]. The disparity in prevalence estimates draws into question the accuracy and purpose of PAE questioning at antenatal appointments. Clinical underreporting may be due to lack of or ineffective questioning and/or maternal concerns regarding anonymity (fear of judgement and/or lack of support from healthcare professional) [172,173,174,175]. The patient’s perception of the importance and/or moral obligation in providing more accurate information for research purposes may also lead to differences between clinic and research estimates. Current tools used for the assessment of PAE in Australian antenatal care include the AUDIT-C, T-ACE, and TWEAK, as mentioned in the current clinical guidelines [176]. The use of AUDIT-C in clinical practice is likely particularly useful, and its application advocated. Recently published findings from a randomized controlled trial suggest that perhaps implementation of this tool could be improved, using three elements of care provision—assess, advise, and refer [177].

## 5. Conclusions

The current study provides the first systematic review and meta-analysis of Australian birth cohort studies that included questions assessing PAE prevalence. Overall, Australia has a high prevalence of prenatal alcohol exposure (48%) in comparison to a previous international meta-analysis that estimated a pooled estimate of 9.8% (CI 8.9–11.1%) globally, although there is distinct geographical deviation [178]. Results demonstrated that compared with the available alcohol-focused studies, PAE prevalence from general cohort studies was lower and more variable. General cohort studies that employed questionnaires or trained interviewers reported higher prevalence estimates that more closely matched alcohol-focused studies. Data collected from clinical settings contained high levels of missing data and lower prevalence estimates compared to research studies, which points to the urgent need for increased training and support for antenatal care providers in assessing PAE and supporting women with alcohol-use concerns.

## Data Availability

The availability of data, code, and other materials (in the form of template data collection forms; data used for all analyses; analytic code; other materials used in the review) is not applicable. The MetaXL used for meta-analysis may be made available online.

## Supplementary Materials

The following supporting information can be downloaded at: https://www.mdpi.com/article/10.3390/ijerph192013144/s1, Table S1: Search terms used; Table S2: Components of the STROBE Statement used as the basis for quality assessment of included cohort studies; Table S3: Components of the EPHPP Quality Assessment tool for Quantitative Studies used as the basis for bias assessment of included cohort studies; Table S4: The final modified tool used to assess quality of reporting and bias in included cohort studies; Table S5: Studies from cohorts included in primary text; Table S6: Questions used to capture alcohol consumption during pregnancy in major cohort studies; Table S7: Cohort characteristics of smaller or more specific cohort studies; Table S8: Summary of prenatal alcohol exposure information collected during pregnancy in smaller or more specific cohort studies; Table S9: Summary of prenatal alcohol exposure information collected during pregnancy in smaller or more specific cohort studies; Table S10: Sensitivity Analysis.
