# Supplementary material for: Prevalence and Patterns of Prenatal Alcohol Exposure in Australian Cohort and Cross-Sectional Studies: A Systematic Review of Data Collection Approaches
Source: Int J Environ Res Public Health. 2022 Oct 12;19(20):13144. doi: 10.3390/ijerph192013144 (PMC9603223; doi:10.3390/ijerph192013144)
Supplement: Supplementary file 1 [file ijerph-19-13144-s001.zip › ijerph-1916069-supplementary.pdf]

Supplementary Material

Table S1. Search terms used

Database	Population	Domain	Cohort	
Search Terms	Maternal OR prenatal OR pre-natal OR neonatal OR neo-natal OR fetal OR foetal OR pregnan*	alcohol OR ethanol OR “fetal alcohol” OR “foetal alcohol”	"Case control" OR cohort* OR "follow up stud*" OR "observational stud*" OR Longitudinal OR Retrospective OR "Cross sectional"	Australia*
MeSH	"Pregnant Women" OR "Pregnancy"	"Alcohol Drinking" OR "Ethanol"	Epidemiologic studies" OR "case control studies" OR "cohort studies" OR "Cross-sectional studies"	"Australia"
CINAHL subject headings	MH “Expectant Mothers” OR MH “Pregnancy+”	MH "Alcohol Drinking+" MH Ethanol+”	MH “Prospective studies” OR MH "case control studies+" OR MH "Cross-sectional studies" OR MH “Correlational studies” OR MH “Nonconcurrent prospective studies”	MH "Australia+"
Emtree	‘Pregnant Woman’/exp OR ‘Pregnancy’/exp	‘Drinking Behavior’/exp OR ‘Alcohol’/exp OR ‘Alcohol consumption’/exp	"case control study”/exp OR "longitudinal study"/exp OR "Cross-sectional study”/exp OR “clinical study”/exp OR “family study”/exp OR “retrospective study”/exp OR “prospective study”/exp OR “cohort analysis”/exp	‘Australia’/exp

[1]Title/Abstract were searched for PubMed, Embase, and CINAHL. Title/Abstract/Key words were searched for Web of Science and Scopus, and ‘all fields’ were searched for Informit.

Table S2. Components of the STROBE Statement used as the basis for quality assessment of included cohort studies

	Item No	Recommendation	Changes
**Title and abstract**	1	(*a*) Indicate the study's design with a commonly used term in the title or the abstract	(*a*) Indicate the study's focus with the term 'alcohol' in the title or the abstract
		(*b*) Provide in the abstract an informative and balanced summary of what was done and what was found	Included
**Introduction**			
Background/rationale	2	Explain the scientific background and rationale for the investigation being reported	Included
Objectives	3	State specific objectives, including any prespecified hypotheses	Included
**Methods**			
Study design	4	Present key elements of study design early in the paper	Included
Setting	5	Describe the setting, locations, and relevant dates, including periods of recruitment, exposure, follow-up, and data collection	Included
Participants	6	(*a*) Give the eligibility criteria, and the sources and methods of selection of participants. Describe methods of follow-up	Included
		(*b*) For matched studies, give matching criteria and number of exposed and unexposed	Removed, as matched studies excluded
Variables	7	Clearly define all outcomes, exposures, predictors, potential confounders, and effect modifiers. Give diagnostic criteria, if applicable	Removed, as the outcome of interest is prevalence
Data sources/ measurement	8*	For each variable of interest, give sources of data and details of methods of assessment (measurement). Describe comparability of assessment methods if there is more than one group	Included
Bias	9	Describe any efforts to address potential sources of bias	Removed as deemed to broad/general
Study size	10	Explain how the study size was arrived at	Included
Quantitative variables	11	Explain how quantitative variables were handled in the analyses. If applicable, describe which groupings were chosen and why	Removed, analyses not applicable
Statistical methods	12	(*a*) Describe all statistical methods, including those used to control for confounding	Removed, analyses not applicable
		(*b*) Describe any methods used to examine subgroups and interactions	Removed, analyses not applicable
		(*c*) Explain how missing data were addressed	Removed, not applicable for analyses and is addressed through question 13
		(*d*) If applicable, explain how loss to follow-up was addressed	Removed, loss to follow up not applicable for analysis
		(*e*) Describe any sensitivity analyses	Removed, sensitivity analysis not applicable
**Results**			
Participants	13*	(a) Report numbers of individuals at each stage of study—eg numbers potentially eligible, examined for eligibility, confirmed eligible, included in the study, completing follow-up, and analysed	Included
		(b) Give reasons for non-participation at each stage	Included, combined with Q1 of 'withdrawals and dropouts' from EPHPP tool
		(c) Consider use of a flow diagram	Included
Descriptive data	14*	(a) Give characteristics of study participants (eg demographic, clinical, social) and information on exposures and potential confounders	(a) Give characteristics of study participants (eg demographic, clinical, social)
		(b) Indicate number of participants with missing data for each variable of interest	Included
		(c) Summarise follow-up time (eg, average and total amount)	Removed, follow up time not relevant as does not changes between participants
Outcome data	15*	Report numbers of outcome events or summary measures over time	Included
Main results	16	(*a*) Give unadjusted estimates and, if applicable, confounder-adjusted estimates and their precision (eg, 95% confidence interval). Make clear which confounders were adjusted for and why they were included	Removed, not applicable as outcome of interest is prevalence
		(*b*) Report category boundaries when continuous variables were categorized	Removed, not applicable as outcome of interest is prevalence
		(*c*) If relevant, consider translating estimates of relative risk into absolute risk for a meaningful time period	Removed, not applicable as outcome of interest is prevalence
Other analyses	17	Report other analyses done—eg analyses of subgroups and interactions, and sensitivity analyses	Removed, not applicable as outcome of interest is prevalence
**Discussion**			
Key results	18	Summarise key results with reference to study objectives	Included
Limitations	19	Discuss limitations of the study, taking into account sources of potential bias or imprecision. Discuss both direction and magnitude of any potential bias	Included

Interpretation	20	Give a cautious overall interpretation of results considering objectives, limitations, multiplicity of analyses, results from similar studies, and other relevant evidence	Included, modified to 'Give a cautious overall interpretation of results considering objectives, limitations, results from similar studies, and other relevant evidence'
Generalisability	21	Discuss the generalisability (external validity) of the study results	Included
**Other information**			
Funding	22	Give the source of funding and the role of the funders for the present study and, if applicable, for the original study on which the present article is based	Included

*Give information separately for exposed and unexposed groups.

Note: An Explanation and Elaboration article discusses each checklist item and gives methodological background and published examples of transparent reporting. The STROBE checklist is best used in conjunction with this article (freely available on the Web sites of PLoS Medicine at http://www.plosmedicine.org/, Annals of Internal Medicine at http://www.annals.org/, and Epidemiology at http://www.epidem.com/). Information on the STROBE Initiative is available at http://www.strobe-statement.org.

Table S3. Components of the EPHPP Quality Assessment tool for Quantitative Studies used as the basis for bias assessment of included cohort studies

Selection Bias	1	Are the individuals selected to participate in the study likely to be representative of the target population?	Included
	2	What percentage of selected individuals agreed to participate?	Included
Study Design	1	Indicate the study design	Removed, as the study design will be similar across studies
	2	Was the study described as randomized?	Removed, no studies will be randomised trials
	3	If Yes, was the method of randomization described?	Removed, no studies will be randomised trials
	4	If Yes, was the method appropriate?	Removed, no studies will be randomised trials
Confounders	1	Were there important differences between groups prior to the intervention?	Removed, no intervention
	2	If yes, indicate the percentage of relevant confounders that were controlled (either in the design (e.g. stratification, matching) or analysis)?	Removed, no intervention
Blinding	1	Was (were) the outcome assessor(s) aware of the intervention or exposure status of participants?	Removed, as in most cases this information is not included
	2	Were the study participants aware of the research question?	Removed, as this applies to 2 cohort studies and so was deemed not relevant
Data Collection Methods	1	Were data collection tools shown to be valid?	Kept, but combined with Q2 in this section: 'Is validity and reliability of the collection tool discussed?'
	2	Were data collection tools shown to be reliable?	Combined with question 1 in this section.
Withdrawals and Dropouts	1	Were withdrawals and drop-outs reported in terms of numbers and/or reasons per group?	Combined with STROBE question 13b.
	2	Indicate the percentage of participants completing the study. (If the percentage differs by groups, record the lowest).	Removed, as this is addressed through STROBE question 13a

Table S4: The final modified tool used to assess quality of reporting and bias in included cohort studies

	Item No	Question	Score	Scoring Comments	Source
**Title and abstract**	1	(*a*) Indicate the study's focus with the term 'alcohol' in the title or the abstract	2	Must include term 'alcohol' in the title for 2; in title or abstract for 1; no = 0	Strobe Q1a, modified
		(*b*) Provide in the abstract an informative and balanced summary of what was done and what was found	2	Yes = 2, partial = 1, no = 0	Strobe Q1b
**Introduction**					
Background/rationale	2	Explain the scientific background and rationale for the investigation being reported	2	Yes = 2, partial = 1, no = 0	Strobe Q2
Objectives	3	State specific objectives, including any prespecified hypotheses	2	Objective and hypotheses = 2; objective or hypotheses = 1; no = 0	Strobe Q3
**Methods**					
Study design	4	Present key elements of study design early in the paper	2	Yes = 2, partial = 1, no = 0	Strobe Q4
Setting	5	Describe the setting, locations, and relevant dates, including periods of recruitment, exposure, follow-up, and data collection	2	Yes = 2, partial = 1, no = 0	Strobe Q5
Participants	6	(*a*) Give the eligibility criteria, and the sources and methods of selection of participants. Describe methods of follow-up	2	Yes = 2, partial = 1, no = 0	Strobe Q6
Data sources/ measurement	7	*(a)* For each variable of interest, give sources of data and details of methods of assessment (measurement) e.g. self-report or interview. Describe comparability of assessment methods if there is more than one group	2	Yes = 2, partial = 1, no = 0	Strobe Q8
		(b) 'Is validity and reliability of the collection tool discussed?'	2	Yes = 2, validity or reliability = 1, no = 0	EPHPP, modified
Study size	8	Explain how the study size was arrived at	2	If included entire population available approached = 2, or explained why the whole available sample was not approached = 1, no explanation = 0.	Strobe Q10
**Selection Bias**					
	9	Are the individuals selected to participate in the study likely to be representative of the target population?	3	If truly representative of Australian sample/population, including participants from a number of different sites and both public and private patients = 3, if mostly representative of Australian population (e.g. large hospital cohort and all invited to participate) = 2, if somewhat representative of Australian population = 1, 'specific' population = 0	EPHPP Q1 of 'selection bias'. Has been modified.
	10	What percentage of selected individuals agreed to participate?	4	>90% agreed to participate or N/A = 4, 80-90 % = 3, 60-80% = 2, less than 60% = 1, not detailed = 0	EPHPP Q2 of 'selection bias'. Has been modified
**Results**					
Participants	11	(a) Report numbers of individuals at each stage of study—eg numbers potentially eligible, examined for eligibility, confirmed eligible, included in the study, completing follow-up, and analysed. Including withdrawals and dropouts.	2	Yes or N/A = 2, partial = 1, no = 0	Strobe Q13a
		(b) Give reasons for non-participation/exclusion at each stage	2	Yes = 2 or N/A, partial = 1, no = 0. N/A if all participate	Strobe Q13b, EPHPP Q1 of 'withdrawals and dropouts' – counted as STROBE in overall estimate
		(c) Consider use of a flow diagram	1	Yes = 1, no = 0	Strobe Q13c
Descriptive data	12	(a) Give characteristics of study participants (eg demographic, clinical, social) and information on exposures	2	Yes = 2, partial = 1, no = 0	Strobe Q14a, modified
		(b) Indicate number of participants with missing data for each variable of interest	2	Yes = 2 or N/A, partial = 1, no = 0. N/A if no missing data	Strobe Q14b
Outcome data	13	Report numbers of outcome events or summary measures of exposure	1	Yes = 1; no = 0	Strobe Q15
**Discussion**					
Key results	14	Summarise key results with reference to study objectives	2	Yes = 2, partial = 1, no = 0	Strobe Q18
Limitations	15	Discuss limitations of the study, taking into account sources of potential bias or imprecision. Discuss both direction and magnitude of any potential bias	2	Yes = 2, partial = 1, no = 0	Strobe Q19
Interpretation	16	Give a cautious overall interpretation of results considering objectives, limitations, results from similar studies, and other relevant evidence	2	Yes = 2, partial = 1, no = 0	Strobe Q20, modified

Generalisability	17	Discuss the generalisability (external validity) of the study results	2	Yes = 2, partial = 1, no = 0. To other similar populations or national population.	Strobe Q21
**Other information**					
Funding	18	Give the source of funding and the role of the funders for the present study and, if applicable, for the original study on which the present article is based	1	Yes = 1, 0 = no	Strobe Q22
Ethics	19	Include an ethics statement	1	Yes = 1, 0 = no	Neither

Table S5. Studies from cohorts included in primary text

Cohort	Included Studies	Reference	Rating STROBE	EPHPP	Ethics	Overall
PEMP	Gibson et al (1983)^	[29]	14	10	1	25
MUSP	O'Callaghan et al (2006)	[39]	25	9	0	34
	Keeping et al (1989)^	[18]				
	Alati et al (2008)	[33]				
	Alati et al (2009)	[34]				
	Alati et al (2006)	[32]				
	Bor et al (2004)	[35]				
	Das et al (2017)	[36]				
	Morrison et al (1993)	[37]				
	O'Callaghan et al (2003)	[38]				
	Salom et al (2015)	[40]				
	Suresh et al (2012)	[41]				
	Tran et al (2015 - Trajectories)	[43]				
	Tran et al (2015 - Predictors)	[42]				
	Kisely et al (2022)	[44]				
Tasmanian cohort study	Correy et al (1991)^	[25]	25	7	0	32
	Kwok et al (1983)	[21]				
	Lumley et al (1985)	[20]				
Victorian cohort study	Bell & Lumley (1989)^	[28]	26	5	0	31
TIHS [1]	Wang et al (2014)	[45]				
	Tikellis et al (2012)^	[19]	26	2	0	28
RAINE	Bhat et al (2017)^	[31]	29	5	1	35
	Grace et al (2017)	[46]				
	Robinson et al (2010 – low-moderate prenatal alcohol)^	[48]				
	Robinson et al (2010 – smoking cessation)	[47]				
	Whitehouse et al (2011)	[49]				
	Ing et al (2021)	[50]				
	Duko et al (2021 – prenatal alcohol)	[51]				
	Duko et al (2022 – prenatal tobacco)	[52]				
	Duko et al (2022 prenatal alcohol)	[53]				

	Duko et al (2021 – prenatal tobacco)	[54]				
	Duko et al (2020)	[55]				
WAPIS (including RASCALS subset) (50, 53, 89-93)	Colvin et al (2007)^	[56]	27	8	0	35
	O'Leary et al (2009 - growth)	[59]				
	O'Leary et al (2009 - language)	[57]				
	O'Leary et al (2010 - behaviour)	[61]				
	O'Leary et al (2010 - birth defects)	[60]				
	O'Leary et al (2010 - fetal effects)	[58]				
	O'Leary et al (2013)	[62]				
LSAC	Sciberras et al (2011)	[67]				
	Guy et al (2016)	[63]				
	Lewis et al (2012 - letters)	[65]				
	Lewis et al (2012)	[66]				
	Hutchinson et al (2013)^	[64]	23	6	1	30
	Hayes et al (2021)	[68]				
	Ahmad et al (2021)	[69]				
	Chandler-Mather et al (2021)	[70]				
ALSWH	Anderson et al (2012)	[73]				
	Anderson et al (2013)^	[71]	32	7	1	40
	Anderson et al (2014)	[72]				
	Powers et al (2010)	[74]				
	Powers et al (2013)	[75]				
NDSHS	Callinan and Ferris (2014)^	[76]				
	Wallace et al (2007)	[77]				
	Maloney et al (2011)	[22]				
SCOPE	Leemaqz et al (2016)	[79]				
	Khomami et al (2019)	[78]				
	O'Keeffe et al (2015)	[80]				
	McCarthy et al (2013)^	[27]	32	5	1	38
EFHL [2]	Cameron et al (2012)^	[30]	30	6	1	37
	Cameron et al (2013)	[81]	22	3	1	26
Triple B	Hutchinson et al (2019)	[84]				
	Hutchinson et al (2018)	[83]				
	Fransquet et al (2016)	[82]				

	McCormack et al (2018)	[85]				
	McCormack et al (2017)^	[10]	30	5	1	36
	Peacock et al (2018)	[86]				
	Rossen et al (2016)	[87]				
AQUA	Muggli et al (2016)	[90]				
	Muggli et al (2014)^	[9]	31	8	1	40
	Muggli et al (2017)	[89]				
	Halliday et al (2017)	[88]				
	Muggli et al (2022 – data driven approach)	[91]				
	Muggli et al (2022 – cohort profile)	[92]				
TBIS	Molloy et al (2020)^	[17]				
	Symeonides et al (2021)	[93]				
	Pham et al (2022)	[94]				
Hunter New England	Tsang et al (2022)	[28]				

^Indicates the primary paper referred to in text. Papers underwent quality evaluation if they contributed to data used in the meta-analysis. [1] Both figures reported the same sample size and prevalence. The rating for the paper published first has been used in the meta-analysis. [2] Where data were combined from two papers for inclusion in the meta-analysis, both papers have been rated.

Table S6. Full text exclusion

Paper	Reason for exclusion
Hendryx, M., et al. (2020). "Latent Class Analysis of Low Birth Weight and Preterm Delivery among Australian Women." Journal of Pediatrics 218: 42-48.e41.	Prevalence not reported. Binge alcohol was examined but coded as 'monthly' or 'less than monthly'.
Hill, B., et al. (2021). "Lifestyle and Psychological Factors of Women with Pregnancy Intentions Who Become Pregnant: Analysis of a Longitudinal Cohort of Australian Women." Journal of Clinical Medicine 10(4)	Pregnancy not examined. Study looked at alcohol use in those with parental aspirations.
Gibson, L. and M. Porter (2018). "Drinking or smoking while breastfeeding and later cognition in children." Pediatrics 142(2).	Prevalence not reported. Study grouped '0' alcohol with 'occasional' so prevalence could not be determined.
Gibson, L. and M. Porter (2020). "Drinking or smoking while breastfeeding and later developmental health outcomes in children." BMC research notes 13(1): 232.	Prevalence not reported. Prenatal alcohol exposure was used as a predictor variable.
Dávila, M. G., et al. (2017). "Maternal Alcohol Consumption and Off spring's Problem Gambling." American Journal of Public Health 107(4): 487-487.	Prevalence not reported. Not a research article.
Hayatbakhsh, M. R., et al. (2007). "Association of maternal smoking and alcohol consumption with young adults' cannabis use: A prospective study." American Journal of Epidemiology 166(5): 592-598.	Pregnancy not examined. Alcohol consumption queried when children aged 5 and 14 years.
Hayatbakhsh, M. R., et al. (2009). "Predictors of young adults' amphetamine use and disorders: a prospective study." Drug & Alcohol Review 28(3): 275-283	Pregnancy not examined. Alcohol consumption queried at 14 year follow up.
O'Callaghan, F. V., et al. (2006). "Prediction of adolescent smoking from family and social risk factors at 5 years, and maternal smoking in pregnancy and at 5 and 14 years." Addiction 101(2): 282-290.	Pregnancy not examined. Alcohol consumption queried at 5 year follow up.
Tran, N. T., et al. (2016). "Gender differences in the prospective association between maternal alcohol consumption trajectories and young adult offspring's problem gambling at 30 years." Asian J Gambl Issues Public Health 6(1): 2.	Prevalence not reported. Study looked at trajectories of maternal alcohol consumption across five time points, and did not specify prevalence of prenatal alcohol exposure during pregnancy.
Peadon, E., et al. (2011). "Attitudes and behaviour predict women's intention to drink alcohol during pregnancy: The challenge for health professionals." BMC public health 11.	Not an infancy or birth cohort. Women were recruited between ages 18-45, with median time since last pregnancy five years (range: less than one year to 23 years).
Odgers, H. L., et al. (2019). "Factors and outcomes associated with an Edinburgh post-natal depression scale score ⩾ 13 at the first antenatal visit." Journal of Paediatrics and Child Health 55: 92-93.	Not a journal article.

Leggat, G., et al. (2021). "Changes in alcohol consumption during pregnancy and over the transition towards parenthood." Drug and Alcohol Dependence 225.	Prevalence not reported.
Doherty, E., et al. (2022). "Practice change intervention to improve antenatal care addressing alcohol consumption during pregnancy: a randomised stepped-wedge controlled trial." BMC Pregnancy and Childbirth 22(1).	Prevalence not reported.

Table S7. Questions used to capture alcohol consumption during pregnancy in major cohort studies[a]

Cohort	Occurrence	Frequency	Quantity	Trimester	Binge/Heavy
**MUSP**		"How often do you drink alcohol since becoming pregnant?"<br>- daily<br>- a few times a week<br>- a few times a month<br>- a few times a year<br>- Rarely<br>- never	How much alcohol do you usually drink at those times?"<br>- Never drink<br>- Less than one glass<br>- One or two glasses<br>- Three or four glasses<br>- Five or six glasses<br>- Seven or more glasses	In the last three months of your pregnancy, how often did you drink alcohol? (asked after delivery)<br>- Daily<br>- a few times a week<br>- a few times a month<br>- not at all	When you drink alcohol what part of the time do you have at least 5 glasses?<br>- Nearly half the time or more<br>- Less than half the time<br>- Never
**Tasmania – General Cohort Study[b]**		Alcohol categories provided, no guidance on question:<br>- Nil<br>- Seldom (Minor intake on social occasions)<br>- Mild or irregular (3-6 drinks weekly)<br>- Moderate (2-3 drinks daily or more)<br>- Heavy (4-5 drinks daily or more)			Alcohol category provided, no guidance on question:<br>- 'Binges' (Heavy intake, possibly irregular)
**Victoria – Bell and Lumley**		How many standard drinks (standard servings of beer or wine or spirits) did the woman			Were there any occasions during pregnancy when she

		have each week during pregnancy?			had five or more drinks in a day?
**TIHS**		Questions for frequency, quantity, trimester, and binge/heavy consumption were combined, with women asked to respond based on their 1st trimester (0-13 wk), 2nd trimester (14-27 wk), and 3rd trimester (28-40 wk). Responses collected on the fourth day of life.<br><br>How much alcohol did you consume during your pregnancy?<br>Drink = 4oz glass white wine, 6-8oz glass beer, ½ nip spirits with mixer.<br>- Nil<br>- 0-1 drink/day<br>- 2-3 drinks/day<br>- 4-5 drinks/day<br>- 6+ drinks/day<br>- More than 5 drinks on any one occasion			
**RAINE**		During the first 3 months of this pregnancy would you say that you drank alcohol (18 weeks)/Would you say that you NOW drank alcohol (34 weeks):<br>- Daily<br>- Several times a week<br>- Approximately once a week<br>- Less than once a week<br>- Never	During the first 3 months of this pregnancy, how many drinks in total did you consume per week (18 weeks)/Would you say that you NOW drank alcohol (34 weeks):<br>[X] glasses of wine<br>[X] nips of spirits<br>[X] cans or stubbies of full strength beer<br>[X] cans or stubbies of low alcohol beer	Asked at 18 weeks what first 3mth consumption was, and at 34 weeks what current consumption was	

**WAPIS**		"Please indicate in each of the following tables, for the four different time periods before and during your pregnancy, how often you drank each of the types of drinks listed…?"<br>- 5 or more days per week<br>- 3-4 days per week<br>- 1-2 days per week<br>- 1-2 days per month<br>- less than once a month<br>- never	"Please indicate …. And how much you drank on a typical occasion?"<br>[open text]<br>Options given: beer, wine/champagne, fortified wines, spirits/liqueurs	"During the 3 months before your recent pregnancy …"<br>"During the first 3 months (1–3 months or 1–13 weeks) of your recent pregnancy …"<br>"During the middle 3 months (4–6 months or 14–26 weeks) of your recent pregnancy …"<br>"During the final 3 months (7–9 months or 27–40 weeks) of your recent pregnancy …"	Binge or heavy could be identified by number consumed on a typical occasion.
**LSAC**	During the pregnancy with the study child, did you drink alcohol?<br>- yes<br>- no	On average, how many days per week did you have a drink during this pregnancy? Days per week in the first three months/middle three months/last three months?<br>- None<br>- 1<br>- 2<br>- 3<br>- 4<br>- 5<br>- 6	On average, about how many standard drinks did you have on the days you did have a drink?<br>- 1 or 2<br>- 3 or 4<br>- 5 or 6<br>- 7 to 10<br>- 11 or more	Days per week in the first three months/middle three months/last three months? (see frequency)	

		- 7<br>- Occasional - not every week			
**ALSWH**		'How often do you usually drink alcohol?'<br>- I never drink alcohol<br>- less than once a month<br>- less than once a week<br>- on 1 or 2 days a week<br>- on 3 or 4 days a week<br>- on 5 or 6 days a week<br>- - everyday	'On a day when you drink alcohol, how many standard drinks do you usually have?'<br>- 1 or 2 drinks per day<br>- 3 or 4 drinks per day<br>- 5 to 8 drinks per day<br>- 9 or more drinks per day	"Are you currently pregnant?"<br>- less than 3 months<br>- 3 to 6 months<br>- more than 6 months<br><br>Women not followed across trimesters.	'How often do you have five or more standard drinks of alcohol on one occasion?'
**NDSHS (2007 and 2010 survey questions shown)**	"At any time in the last 12 months when you were pregnant or breastfeeding, did you use any of the following?" (alcohol as option)<br>- When pregnant only<br>- When breastfeeding only<br>- When pregnant and breast-feeding<br>(2010) At any time in the last 12 months when you were pregnant or breastfeeding, did you use any of the following? (alcohol as option)		(2010) In the last 12 months when you were pregnant, in general, did you drink more, less or the same amount of alcohol compared to when you were neither pregnant nor breastfeeding? (Mark one response only)<br>- More<br>- Less<br>- Same amount<br>- Don't drink alcohol		

	- Before knowledge of pregnancy<br>- After knowledge of pregnancy<br>- When breastfeeding		- Not applicable, was not pregnant in the last 12 months		
**SCOPE**	Were you drinking alcohol before pregnancy?<br><br>Were you drinking alcohol earlier in the pregnancy?<br><br>Are you still drinking alcohol?				
**EFHL**	Have you had an alcoholic drink of any kind during your pregnancy?<br>- Yes<br>- No	During your pregnancy, how often did you have an alcoholic drink of any kind?<br>- Every day<br>- 5 to 6 days a week<br>- 3 to 4 days a week<br>- 1 to 2 days a week<br>- 2 to 3 days a month<br>- About 1 day a month<br>- Less often<br>- No longer drink	On a day that you have an alcoholic drink, how many standard drinks do you usually have (picture reference of standard drinks guide provided)<br>- 13 or more drinks<br>- 11 to 12 drinks<br>- 7 to 10 drinks<br>- 5 to 6 drinks<br>- 3 to 4 drinks<br>- 1 to 2 drinks	From 2007 onwards, participants were asked to indicate their responses to frequency, quantity, and binge/heavy exposure questions for each time period (early pregnancy 0-13 wks, mid pregnancy 14-26 wks, late pregnancy 27-42 wks).	(2006) During your pregnancy, how often did you have 5 or more standard drinks on one occasion?<br>- Every day<br>- 5 to 6 days a week<br>- 3 to 4 days a week<br>- 1 to 2 days a week<br>- 2 to 3 days a month<br>- About 1 day a month<br>- Less often<br>- Never

**TBIS**			During the 1st & 2nd trimesters of your pregnancy, how many standard alcoholic drinks have you consumed? (Please tick one box for each time period)<br>- no standard drinks,<br>- 1 per week,<br>- between 1 and 6 per week,<br>- 1 per day)<br><br>How many standard alcoholic drinks have you had in total over the 1st trimester (0-13 weeks)?<br><br>How many standard alcoholic drinks have you had in total over the 2nd trimester (14-27 weeks)?<br><br>How many standard alcoholic drinks have you had in the last 4 weeks?<br>- no standard drinks,<br>- 1 per week,<br>- between 1 and 6 per week,<br>- 1 per day		How many times did you drink >5 standard drinks on any one occasion (e.g. in the space of a few hours) during your 1st trimester (0-13 weeks)?<br><br>How many times did you drink >5 standard drinks on any one occasion (e.g. in the space of a few hours) during your 2nd trimester (14-27 weeks)?
**Hunter New England**		How often do you have a drink containing alcohol? (SCORE)<br>- Never (0)<br>- Monthly or less (1)<br>- Two to four times a month (2)<br>- Two to three times per week (3)<br>- Four or more times a week (4)	How many drinks containing alcohol do you have on a typical day when you are drinking?<br>- 1 or 2 (0)<br>- 3 or 4 (1)<br>- 5 or 6 (2)<br>- 7 to 9 (3)<br>- 10 or more (4)		How often do you have six or more drinks on one occasion?<br>- Never (0)<br>- Less than Monthly (1)<br>- Monthly (2)<br>- Two to three times per week (3)<br>- Four or more times a week (4)

					Participants were also asked about alcohol consumption during special occasions after pregnancy recognition

[a] Where full questionnaire could not be found but some detail was provided, this detail has been included in table. [b] Form included space for quite date (weeks) of prenatal alcohol, if relevant.

Table S8. Cohort characteristics of smaller or more specific cohort studies

Cohort	Number enrolled in study*	Year of enrolment	Original questionnaire details available/Method of collection	Reason for exclusion from main paper
**General Cohorts**				
DADHI - Singh [95]	82	Unclear	No/Unclear	<1000
Adelaide – Douglas [96]	836	1987-1988	No/Interview	Sample size <1000
Aus-Acute Lymphoblastic Leukemia [97]	1028	2003-2007 (1988-2006)	No/Self-administered questionnaire	Case control study
Adolescent pregnancy [98]	537	1997-1999	No/Unclear	<1000
Adolescent pregnancy [99]	160	1997-1998	No/Unclear, likely interview	<1000
Adolescent pregnancy [100]	456	1998-2000	No/Unclear, likely interview	<1000
Adelaide – Hotham [101]	748	2005-2006	No/Self-administered questionnaire	Sample size <1000
Antepartum risk for newborn encephalopathy [102]	564	1993-1995	No/Self-administered questionnaire	Sample size <1000, case control study
Autism Spectrum Disorder[103]	137	-	Yes/Self-administered questionnaire	Sample size <1000, not a general antenatal population
Cairns – Gilligan [104]	388	2006	No/Interview	<1000
Adelaide – Malek [105]	857	2013	No/Self-administered questionnaire (online)	<1000
Canberra – Behie [106]	53	2018 (pregnancy date unclear)	Yes/Interview	Sample size <1000
WA Burman – Case control [107]	265	1982-1984	No/Self-administered questionnaire	Case control study
Aus-Childhood Brain Tumour [108]	1019	2005-2010 (1990-2010)	No/Self-administered questionnaire	Case control study
Combo Aus-ALL, Aus-CBT [109]	2086	2003-2010 (1988-2010)	No/Self-administered questionnaire	Case control study
Drug using mothers [110]	871	2004	No/Likely interview (data linkage)	Drug/alcohol use disorder, sample size <1000,
HepC – O'connor [111]	570	2009-2012	No/Likely interview (data linkage)	Drug/Alcohol use disorder, sample size <1000, data linkage.
Dopamine – Oei [112]	97, but 49 of these were abstainers (control)	1999-2006	No/Likely interview	Drug/alcohol use disorder, sample size <1000.
BM or MM – Whitham [113]	85	2002-2006	No/Interview	Sample <1000.
Epilepsy [114]	992	1999-2006	No/Interview	Sample <1000, not a general antenatal population.
HSHK [115]	935	2009-2010	No/Interview	Sample <1000.
GDM-Galbally [116]	539	-	No/Likely interview	Sample <1000, data linkage
Schizophrenia [117]	98	2007-2016	No/Interview	Sample <1000, not a general antenatal population.
Severe mental illness – Nguyen [118]	138	2007-2011 .	No/Interview	Sample <1000, not a general antenatal population
Online survey – kennedy [119]	9483	2011-2012	No/Self-administered questionnaire (online)	Australian sample <1000, Australia only figures not given.
Online survey – Zammit [120]	248-200 from Aus	Sometime between 2001-2007	No/Self-administered questionnaire	Australian sample <1000.
GDM – Moran LIMIT study [121]	291	2008-2010	No, but does list questionnaire – The Harvard Willett/Self-administered questionnaire	Non-general antenatal population, sample size <1000.
PIFS (II) [122,123]	587	2002-2003	No/self-administered baseline questionnaire, follow-up telephone interview	Sample <1000.
Sydney – Kesby [124]	99	? prior to 1991	No/Interview	Sample <1000
Victoria – Lumley [125]	292	1989	No/Self-administered questionnaire (postal)	Sample <1000
Victoria – McCarty [126]	143	Unclear	No/self-administered questionnaire	Case control, sample size <1000.

Victoria- Skaren [127]	354	2008-2009	No/unclear, likely self-administered questionnaire	Sample size <1000
Victorian Intergenerational Health Cohort Study [128,129]	398	2006-2013	Telephone interview	Sample size <1000
Australian Temperament Project (Generation 3) [128]	393	2012-2018	Telephone interview	Sample size <1000
WA – Silva [130]	321	2009-2010 (Unclear. Mean age 12.9 so likely around 1996/7.)	No/Self-administered questionnaire	Not general antenatal population, sample size <1000.
The Maternal Health in Pregnancy Study [131-133]	665 [131-133]	1982-1984	No – some detail available though, says first author can be contacted for access/self-administered questionnaire	Sample size <1000
McMahon (Assisted Reproductive Technology) [134]	512	Unclear	No/Unclear, likely interview	Non-general antenatal population, sample size <1000, not likely to be representative of general population (0% prevalence).
Rural Infant Feeding Study [135]	489	April 2010 – November 2011	No/Self-administered questionnaire	Non-general antenatal population, sample size <1000
Every-day activities in pregnancy [19]	576	August 2008 – April 2009	No/Self-administered questionnaire	Sample size <1000
Indigenous Cohort				
Bibbulung Gnardeep [136]	273	15month period in mid to late 1990s	No – assessed via table where F/Q indicated for diff types of alcohol/interview	Sample size <1000, not a general antenatal population.
Substudy of ABCD NRP- Gausia [137]	457	2010-2012	No/n.a.	Data linkage, sample size <1000, not a general antenatal population.
Humphrey [138,139]	96	prior to 2000	No/Likely interview	Sample size <1000, not a general antenatal population
Panaretto [140]	456	2000-2003	No/Likely interview	Sample size <1000, not a general antenatal population.
Passey [141]	257	2009-2011	No, does give: 'any alcohol in the previous month (never, only once, 2 - 4 times in the month, 2 - 3 times a week, or ⩾4 times a week)'./Interview	Sample size <1000, not a general antenatal population.
Reeve [142]	155	2007-2011	No/Interview	Sample size <1000, not a general antenatal population, partial data linkage.
LSIC [143]	1671	2008	No/Interview	Not a general antenatal population, data linkage
Marulu FASD prevention study [144]	586	2008-2015	No/Interview	Sample size <1000, not a general antenatal population
Lililwan Project [18]	134	2010-2011	Yes/Interview	Not a general antenatal population, sample size <1000
Data Linkage				
Humphrey [145]	16176	1992-2001	No/Interview	Data linkage
O'Callaghan [146]	1741	2008-2010	No/Unclear	Data linkage
Kingsbury [147]	19699	2001-2006	No/Interview	Data linkage
Zhao [148]	22,193	2009-2011	No/Interview	Data linkage
Liu [149]	69253	2010-2017	No/Interview	Data linkage, case control
Ng [150]	1857	2005-2014	No/Interview	Data linkage
Shipstone et al [151]	228	2010-2014	No/Unclear	Data linkage, sample size <1000

Table S9. Summary of prenatal alcohol exposure information collected during pregnancy in smaller or more specific cohort studies

Cohort	Prior to conception		Prior to pregnancy recognition		During pregnancy							
	Examined	Prevalence	Examined	Prevalence	Occurrence	Frequency	Quantity	Heavy	Binge	Prevalence	Trimesters	Quit Date
General Cohorts												
DADHI [P]- Singh [95]	-	-	-	-	X	-	-	-	-	59.6%	-	-
Adelaide – Douglas[R] [96]	-	-	-	-	X	X	X	-	-	27.2%, 26.3% <1/day, 0.9% >1/day in trimester 3	-	-
Aus-Acute Lymphoblastic Leukemia[R] [97]	X	-	-	-	X	-	-	-	-	32.5% (ALL), 42.9% (Control), overall: 39.53%	-	-
Adolescent pregnancy[P][98]	-	-	-	-	X	-	-	-	-	19.1% control, 39.1% DV, 24.9% overall	-	-
Adolescent pregnancy – 3[P] [99]	-	-	-	-	X	-	-	-	X	Control group n=60, 13%. Assessed n=100, 21%, 5% binge drinking. Overall n=160, 18%	-	-
Adolescent pregnancy -2[P] [100]	-	-	-	-	X	-	-	-	-	13% non-drug users (n=363), 39% marijuana group (n=62), 52% multidrug group (n=31). Overall 19.2% n=456	-	-
Adelaide – Hotham[P] [101]	X	89.2%	-	-	X	X	X	X	X	11.9% overall, 11.5% for those enrolled T1, 12.5% enrolled T2, 15.6% T3.	-	X
Antepartum risk for newborn encephalopathy [102]	-	-	-	-	X	-	-	-	-	35.3% overall, 30.6% those with encephalopathy, 29.2% control	-	-
Autism Spectrum Disorder [103]	-	-	-	-	X	-	-	-	-	27% at any time, 19.7% T1, 2.2% T2, 5.1% T3	X	-
Cairns – Gilligan[P] [104]	X	33% (low vulnerability n=194), 26% (high vulnerability n=85), 35% (moderate vulnerability n=109). Overall: 31.8%	-	-	X	X	X	X	X	Low vulnerability: 2%, high vulnerability 12%, moderate vulnerability 3%. 11% binge.T3.	-	-
Adelaide – Malek[P,] [105]	-	-	-	-	X	-	-	-	-	21%	-	-
Canberra – Behie[R] [106]	-	-	-	-	X	X	X	X	-	16%	X	-
WA Burman – Case control[R] [107]	-	-	-	-	X	-	-	-	-	47% (control), 48% (left lip/palate). Overall: 47.3% T1	-	-
Aus-Childhood Brain Tumour[R] [108]	-	-	-	-	X	-	-	-	-	30.4% (CBT)-39.9% (Control). Overall: 37.2%	-	-
Combo Aus-ALL, Aus-CBT[R] [109]	X	63.4% ALL mothers, 76.3% ALL control mothers, 69.7% CBT case mothers, 76.6% CBT control mothers			X	X	X	X	-	30.4% ALL case mothers, 40.6% ALL control mothers, 30.3% CBT case mothers, 38.5% CBT control mothers. Overall:37.5%	X	-
Drug using mothers [110]										15.5%		
HepC – O'connor[R] [111]	-	-	-	-	X	-	-	-	-	60% HepC negative, 35% HepC positive. 49% overall	-	-
Dopamine – Oei[R] [112]	-	-	-	-	X	-	-	-	-	8.7% (no neonatal abstinence syndrome) - 11.5% (neonatal abstinence syndrome). No % for control.	-	-
BM or MM – Whitham[P] [113]	-	-	-	-	X	X	X	*Excluded*	-	At enrolment: 42% (control), 50% (Buprenoprhine), 29% (Methadone). Any	-	-

										stage in pregnancy: 64% (control), 50% (buprenorphine), 55% (methadone).		
Epilepsy[P] [114]	-	-	-	-	*X*	-	-	-	-	26.9% overall,	-	-
HSHK[R] [115]	-	-	-	-	*X*	-	-	-	-	*9.8%*	-	-
GDM-Galbally[R] [116]	-	-	-	-	*X*	-	-	-	-	6.8% (GDM) -14.6% (non-GDM).	-	-
Schizophrenia[R] [117]	-	-	-	-	*X*	*X*	-	-	-	12.3% (no psychiatric admission in pregnancy n=40), 44.1% (psychiatric admission n=58)	-	-
Severe mental illness – Nguyen[R] [118]	-	-	-	-	*X*	-	-	-	-	19.6%	-	-
Online survey – kennedy[P/R] [119]	-	-	-	-	*X*	-	-	-	-	23.7% (use of herbals), 76.3% (no use of herbals)	-	-
Online survey – Zammit[P] [120]	*X*	*85.9%*	*X*	*64.1% pre-confirm*	*X*	*X*	*X*	*X*	*X (asked to mark special events)*	67.3% post confirm, 76.6% at any stage	-	-
GDM – Moran LIMIT study[P] [121]	-	-	-	-	*X*	*?*	*X*	*?*	*45.7% (10-20wk), 16.5% 28 weeks, 16.8% 36 weeks.*	?	*X*	-
PIFS (II)[R] [122,123]	*X*	*77% [123]*	-	-	*X*	-	-	-	-	*35.6% [152]*	-	-
Sydney – Kesby[R] [124]	-	-	*X*		*X*	*X*	*X*	*X*	-	43% (T1 post-confirmation), 60% (T2), 65% (T3)	*X*	-
Victoria – Lumley[R] [125]	-	-	-	-	*X*	*X*	*X*	*X*	-	36.2% (non-attenders of childbirth education), 53.5% (attended childbirth class), 50.72% overall	-	-
Victoria – McCarty[R] [126]	-	-	-	-	*X*	*X*	*X*	*?*	*?*	Strabismus: 42% (T1), 33% (T2), -40% (T3). Control: 53% (T1), 33% (T2), 44% (T3). Overall: 49.7% (T1), 33% (T2), 42.8% (T3).	*X*	-
Victoria- Skaren[P] [127]	-	-	-	-	*X*	-	-	-	-	20.9%	-	-
Victorian Intergenerational Health Cohort Study[P] [128,129]	X	-	*X*	76%	*X*	*X*	*X*	*X*	*X (≥4 SD per occasion)*	24% post-recognition	*X (T3)*	-
Australian Temperament Project (Generation 3)[P][128]	X	-	*X*	77%	*X*	*X*	*X*	*X*	*X*	12% post-recognition	*X (T3)*	-
WA – Silva[R] [130]	*X*	*39.9% (3 months before ± during pregnancy), 63.9% (3 month before pregnancy (unplanned) ± during pregnancy)*	-	-	*X*	-	-	-	-	29.3%	-	-
The WA Maternal Health in Pregnancy Study[P] [131-133]	*X*	-	-	-	*X*	*X*	*X*	*X*	*X*	78.2% [132]. Unclear as did not separate 'abstinent' from 'low' in table	-	-

McMahon (Assisted Reproductive Technology)$^{P/R}$. [134]	-	-	-	-	X	X	X	-	-	0%	-	-
Rural Infant Feeding Study [135]	X	84.1%	-	-	X	X	X	X	X	19.9%	-	-
Every-day activities in pregnancy [19]	-	-	-	-	X	-	-	-	-	20.6%	-	-
Indigenous Cohorts												
Bibbulung Gnardeep^{R} [136]	-	-	-	-	X	X	X	X	-	*Not given*	-	-
Substudy of ABCD NRP-Gausia^{P} [137]	-	-	-	-	X	-	-	-	-	33%	-	-
Humphrey^{P} [138,139]	-	-	-	-	X	X	X	X	-	*38.5% indigenous, 18.5% caucasian*	-	-
Panaretto^{P} [140]	-	-	-	-	X	X	X	X	-	27.0%	-	-
Passey^{P} [141]	X	55%	X	51.8%	X	X	-	-	-	*21% current use*	-	X
Reeve^{P} [142]	-	-	-	-	X	-	-	-	-	45%	-	-
LSIC^{R} [143]	-	-	-	-	X	-	-	-	-	22%	-	-
Marulu FASD prevention study [144]	-	-	-	-	X	-	-	-	-	*40.8% overall, 43.2% T1 (of those where T1 data available)*	X	-
Lililwan Project^{R} [18]	X	71%	-	-	X	X	X	X	X	55%	X	-
Data Linkage												
Humphrey$^{R\ (P\ collection)}$ [145]	-	-	-	-	X	-	-	-	-	*22.5% (calculated)*	-	-
O'Callaghan^{R} [146]	-	-	-	-	X	X	X	-	-	-	-	-
Kingsbury^{P} [147]	**X**	25.4%	-	-	X	X	X	X	-	*5.9% drank weekly*	-	-
Zhao^{P} [148]	-	-	-	-	X	-	-	-	-	0.8%	-	-
Liu$^{R\ (P\ collection)}$ [149]	-	-	-	-	X	-	-	-	-	*4.4% No CHD, 7.0% CHD, 10.0% TGA, 7.6% septal, 6.0% RHL, 3.8% LHL, 7.9% Other. Overall: 4.42%*	-	-
Ng^{P} [150]	-	-	-	-	X	-	-	-	-	1.29%	-	-
Shipstone [151]	-	-	-	-	X	-	-	-	-	*12.6% overall, 21.7% indigenous, 9.6% non-indigenous*	-	-

Table S10. Sensitivity Analysis

Excluded Study	Pooled Prevalence	LCI 95%	HCI 95%	Cochran Q	P	I^2	I^2 LCI 95%	I^2 HCI 95%
PEMP	0.461	0.388	0.535	2804.099	0.000	99.608	99.542	99.664
MUSP	0.475	0.368	0.583	5722.511	0.000	99.808	99.783	99.829
Tasmania Cohort Study	0.504	0.410	0.597	4849.237	0.000	99.773	99.742	99.800
Victorian Cohort Study	0.485	0.380	0.591	5535.46	0.000	99.801	99.776	99.824
THIS	0.486	0.383	0.589	5374.896	0.000	99.795	99.769	99.819
RAINE	0.478	0.378	0.578	5805.444	0.000	99.811	99.787	99.832
WAPIS	0.470	0.371	0.570	5409.609	0.000	99.797	99.770	99.820
LSAC	0.483	0.381	0.585	5709.314	0.000	99.807	99.783	99.829
ALSWH	0.461	0.370	0.553	4902.510	0.000	99.776	99.745	99.802
SCOPE	0.483	0.385	0.581	5798.029	0.000	99.810	99.786	99.832
EFHL	0.460	0.333	0.589	5816.305	0.000	99.811	99.787	99.832
Triple B	0.471	0.374	0.568	5638.607	0.000	99.805	99.780	99.827
AQUA	0.470	0.374	0.568	5643.971	0.000	99.805	99.780	99.827
